# A best practice fall prevention exercise program to improve balance, strength / power, and psychosocial health in older adults: study protocol for a randomized controlled trial

**DOI:** 10.1186/1471-2318-13-105

**Published:** 2013-10-09

**Authors:** Yves J Gschwind, Reto W Kressig, Andre Lacroix, Thomas Muehlbauer, Barbara Pfenninger, Urs Granacher

**Affiliations:** 1 Basel Mobility Center, University Hospital Basel, University Center for Medicine of Aging Basel, Schanzenstrasse 55, Basel, 4031, Switzerland; 2 University of Basel and Felix Platter-Hospital Basel, University Center for Medicine of Aging Basel, Basel, Switzerland; 3 Faculty of Human Sciences, Department of Training and Movement Sciences, University of Potsdam, Am Neuen Palais 10, Potsdam, 14469, Germany; 4 Swiss Council for Accident Prevention (bfu), Hodlerstrasse 5a, Bern, 3011, Switzerland

**Keywords:** Seniors, Fall risk assessment, Resistance training, Postural stability

## Abstract

**Background:**

With increasing age neuromuscular deficits (e.g., sarcopenia) may result in impaired physical performance and an increased risk for falls. Prominent intrinsic fall-risk factors are age-related decreases in balance and strength / power performance as well as cognitive decline. Additional studies are needed to develop specifically tailored exercise programs for older adults that can easily be implemented into clinical practice. Thus, the objective of the present trial is to assess the effects of a fall prevention program that was developed by an interdisciplinary expert panel on measures of balance, strength / power, body composition, cognition, psychosocial well-being, and falls self-efficacy in healthy older adults. Additionally, the time-related effects of detraining are tested.

**Methods/Design:**

Healthy old people (n = 54) between the age of 65 to 80 years will participate in this trial. The testing protocol comprises tests for the assessment of static / dynamic steady-state balance (i.e., Sharpened Romberg Test, instrumented gait analysis), proactive balance (i.e., Functional Reach Test; Timed Up and Go Test), reactive balance (i.e., perturbation test during bipedal stance; Push and Release Test), strength (i.e., hand grip strength test; Chair Stand Test), and power (i.e., Stair Climb Power Test; countermovement jump). Further, body composition will be analysed using a bioelectrical impedance analysis system. In addition, questionnaires for the assessment of psychosocial (i.e., World Health Organisation Quality of Life Assessment-Bref), cognitive (i.e., Mini Mental State Examination), and fall risk determinants (i.e., Fall Efficacy Scale – International) will be included in the study protocol. Participants will be randomized into two intervention groups or the control / waiting group. After baseline measures, participants in the intervention groups will conduct a 12-week balance and strength / power exercise intervention 3 times per week, with each training session lasting 30 min. (actual training time). One intervention group will complete an extensive supervised training program, while the other intervention group will complete a short version ('3 times 3’) that is home-based and controlled by weekly phone calls. Post-tests will be conducted right after the intervention period. Additionally, detraining effects will be measured 12 weeks after program cessation. The control group / waiting group will not participate in any specific intervention during the experimental period, but will receive the extensive supervised program after the experimental period.

**Discussion:**

It is expected that particularly the supervised combination of balance and strength / power training will improve performance in variables of balance, strength / power, body composition, cognitive function, psychosocial well-being, and falls self-efficacy of older adults. In addition, information regarding fall risk assessment, dose–response-relations, detraining effects, and supervision of training will be provided. Further, training-induced health-relevant changes, such as improved performance in activities of daily living, cognitive function, and quality of life, as well as a reduced risk for falls may help to lower costs in the health care system. Finally, practitioners, therapists, and instructors will be provided with a scientifically evaluated feasible, safe, and easy-to-administer exercise program for fall prevention.

**Trial registration:**

ClinicalTrials.gov Identifier: NCT01906034

## Background

Worldwide, the number of people over 60 years is growing faster than any other age group and expected to grow from 688 million in 2006 to almost 2 billion by 2050 [1]. The main reasons for this substantial demographic change are higher life expectancy and declining birth rates [2]. This future increase in the proportion of older adults is important from a public health perspective [3]. Aging is generally associated with progressive decline in physical and psychological health [4,5], increased risk of disability and dependency [4], as well as an increase in the number of comorbidities [6]. This decrease in health status is mainly responsible for one of the most common and serious public health problems, namely falls. Over 33% of community-dwelling people aged over 65 years fall at least once a year, and of those 50% will have recurrent falls [7,8]. With increasing age, the rate of falls can increase up to 60% [7,9]. Older adults suffering from cognitive decline may fall twice as often compared to their healthy counterparts [10], while institutionalized older adults in nursing homes or old people’s homes fall even more often [11].

Despite frequent falling in older adults, only one in five falls requires medical attention while less than 10% lead to a fracture [12]. However, in terms of morbidity and mortality, injurious falls have serious consequences of which the hip fracture is the most feared one [13]. Hip fractures often affect functionality and autonomy of older adults [14], and are associated with an overall mortality of 22% to 29% one year after injury [15]. In this context, 27% of older adults require a walking aid one year after a hip fracture surgery [16]. Despite rehabilitation, many individuals do not regain the level of functional performance they had before the fracture [14] which is why fall prevention is important.

Detection of fall risk factors is essential to implement effective and specifically tailored fall prevention strategies [17]. Some fall risk factors are irreversible while others are potentially modifiable with appropriate interventions [18,19]. Regularly conducted objective, reliable and valid fall risk assessment protocols can assist in identifying individuals at risk to make recommendations and optimize prevention strategies [20]. Three of the most common modifiable intrinsic (subject-related) fall risk factors are muscle weakness (relative risk ratio / odds ratio 4.4), balance deficits (relative risk ratio / odds ratio 2.9), and gait instabilities (relative risk ratio / odds ratio 2.9) [9,19,21]. These intrinsic risk factors may be modified by exercise referred to as structured, planned and repetitive physical activities in community-based organized exercise programs [22,23].

Balance is important for maintaining postural equilibrium and thus for the avoidance of falls. Aging may affect central nervous system (i.e., changes in brain volume) and neuromuscular system properties (i.e., loss of sensory and motor neurons) leading to deficits in balance and gait performance [24]. According to Shumway-Cook and Woollacott [25] balance can be subdivided into static / dynamic steady-state (i.e., maintaining a steady position in sitting, standing and walking), proactive (i.e., anticipation of a predicted disturbance), and reactive (i.e., compensation of a disturbance) balance [26,27]. Recently, Muehlbauer et al. [26] were able to show that there is no significant association between measures of steady-state, proactive, and reactive balance in healthy older adults. Thus, for testing and training purposes, balance tests and exercises should target all three domains separately and additionally include dual or multi tasks situations [26], given that multi-tasking is required for the performance of many activities of daily living (ADL, e.g., walking downstairs while talking on the phone) [28,29]. Furthermore, specific balance exercises may help to counteract balance deficits and gait instabilities by reducing the risk of falls in older adults [30-33].

Besides balance, muscle strength / power is required for the successful performance of ADL [26]. General causes of age-related skeletal muscle mass loss (i.e., sarcopenia) are manifold (e.g., cellular, neural, metabolic, hormonal contributors) [5,34,35]. For the diagnosis of age-related sarcopenia the European Working Group on Sarcopenia in Older People (EWGSOP) recommends using the criteria low muscle mass plus either low muscle strength or low physical performance measured by gait velocity (≤80 cm/s), grip strength and muscle mass [36]. Humans loose approximately 20% to 30% of their skeletal muscle mass between young adulthood and 80 years of age [37]. This loss in muscle fibre size and number predominantly occurs in type II muscle fibers which lead to a more rapid decline in muscle power compared to overall muscle strength [38]. This is detrimental because muscle power is an important prerequisite for quick postural reactions in response to external perturbations [39]. Older adults often use the hip or step strategy when balance is threatened [7,32]. A decrease in muscle power would delay such postural reactions to external perturbations [40,41], probably leading to a loss of balance [42] and ultimately resulting in a fall [7].

Based on a thorough fall-risk assessment, specifically tailored balance and resistance training programs can be developed which have the potential to improve important intrinsic fall-risk factors like deficits in muscle strength / power and balance performance [27]. For fall prevention, exercises for the promotion of static / dynamic steady-state, proactive and reactive balance should be trained complementarily [43]. Progression during training can be achieved by reducing the base of support (e.g., bipedal, step, tandem, monopedal stance) and by diminishing the sensory input (e.g., exercises with eyes opened / closed; exercises on stable / unstable surfaces) [21,44]. Additionally, resistance training with a focus on muscle strength / power for the lower extremities and the trunk muscles [45] seems essential for counteracting intrinsic fall risk factors (i.e., muscle weakness) in older adults.

During the past decades, many fall prevention interventions have proven a positive effect of exercise on intrinsic fall risk factors [12]. Despite substantial evidence, these programs have not been sufficiently implemented into clinical practice [46]. To reduce the burden of falls in older adults, easy-to-administer fall prevention programs need to be developed and implemented nationwide. However, lack of skilled people, inadequate communication between researchers, policy makers and clinicians, and health system barriers including inadequate financial resources hinder the implementation of new research evidence into practice [46,47]. Besides a lack of evidence about how fall prevention can be incorporated into community services [48], there is hardly any data available regarding dose–response relationships for optimal exercise for fall prevention. Hence, the Swiss Council for Accident Prevention (bfu) convened an international expert panel (n = 8) consisting of geriatricians, physiotherapists, and health, sports, exercise, accident and fall prevention scientists to conceptualize optimal resistance and balance training programs for fall prevention in older adults. The professional knowledge of the expert panel, the framework of the Manual for Falls Prevention Classification System from the Prevention of Falls Network Europe (ProFaNE) and recent state-of-the-art research, especially in a Swiss context, built the basis for the production of a cost-free practice guide open to the public (available in German or French: http://www.stuerze.bfu.ch) [12,47,49].

The proposed trial presented in this article will investigate the effects of a fall prevention exercise program developed by an expert panel on intrinsic fall risk factors (i.e., balance, strength / power), body composition, cognitive function, psychosocial well-being, and falls-self efficacy. The applied research tools will allow diagnosis of sarcopenia according to the EWGSOP guidelines. Thus, we will be able to evaluate prevalence of sarcopenia in our participants, and conduct sensitivity and specificity analysis for the strength / power assessments including their cut-offs. To facilitate transfer into clinical practice, simple clinical tests for each instrumented test will be provided to alleviate fall risk assessment and exercise prescription adjustment. In addition to an easy implementation into practice, this will allow cross-validation of the applied research instruments (clinical vs. instrumented). Further, this work may help to promote the protocol of the expert panel and the rationale behind the practice guide to people with English as their native language. We hypothesize that our training program will positively influence balance, strength / power, body composition as well as cognition, psychosocial well-being, and falls-self efficacy in older community-dwelling people.

## Methods/Design

### Participants

**Figure 1:**
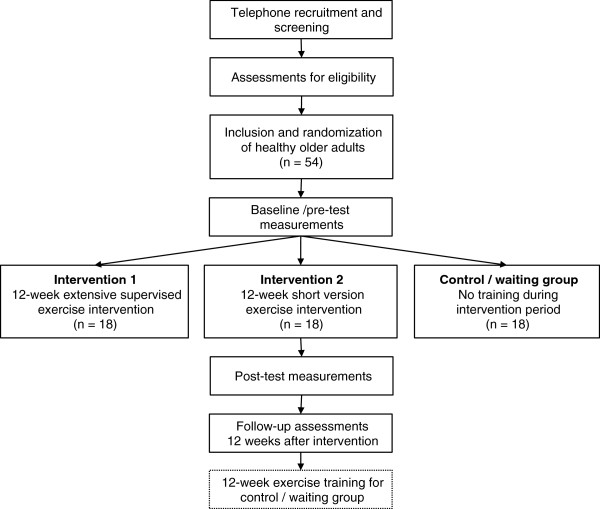
Flow chart of study design.

Community-dwelling older adults aged 65 to 80 years without neurophysiologic diseases will be included in this single centre, randomised, controlled study. Figure 1 shows a flow chart of the study design. Eligibility will be screened with the Standard Assessment Protocol of the Acute Geriatrics Department at the University Hospital Basel / Felix Platter-Hospital Basel including demographic, anthropometric and medical data to rule out contraindications to exercise. Participants will be excluded when they reach cut-off scores for the following tests: Mini Mental State Examination score (MMSE, <24 points) [50,51], Clock Drawing Test (CDT, pathological test performance) [52], Tuning Fork test (individual vibration threshold) [53], Falls Efficacy Scale – International (FES-I, >24 points) [54], World Health Organisation Quality of Life Assessment-Bref (WHOQOL-Bref) [55], and the Freiburg Questionnaire of Physical Activity (FQoPA, less than 1 hour of everyday and sports-related physical activity per week) [56]. Evidence showed that even sedentary older adults are not at increased risk for injury when performing an exercise program compared to young adults [57]. Written informed consent will be obtained from all older adults prior to inclusion. This study is approved by the ethics committee of the University of Potsdam (reference number 34/2012), Germany, and will be conducted according to the ethical standards of the Helsinki Declaration.

### Questionnaires

#### Clock drawing test (CDT)

The CDT will be used for cognitive screening [58]. Participants will be asked to “Please draw a clock and write all the numbers and hands” on a pre-drawn circle of 10 cm in diameter. Afterwards they will be instructed to “Write down the time your clock shows as if it were in a schedule for trains or in a TV guide”. The CDT will be graded pathological if any mistakes in writing the numbers and hands, or writing down the time occur. Inter-rater reliability was shown to be high (IRR = .909) [52].

#### Falls efficacy scale – international version (FES-I)

Falls self-efficacy will be measured using the German 16-item FES-I [54]. This questionnaire measures the level of concern about falling during social and physical activities indoors and outdoors on a 4-point Likert scale (1 = not at all concerned to 4 = very concerned). Internal validity (Cronbach’s alpha = .96) and test-retest reliability (ICC = .96) have been shown to be excellent [59]. Additionally, a 12-months fall history will be collected at baseline.

#### World health organisation quality of life assessment-bref (WHOQOL-Bref)

Quality of life and general health will be assessed by 26 items on a 5-point Likert scale in four domains: physical health, psychological health, social relationship and environment [60]. Scores for the WHOQOL-Bref range from 0–100 with a higher score indicating better quality of life. For this study, the German version of the WHOQOL-Bref will be applied [61]. The WHOQOL-Bref performs according to international standards in terms of reliability, validity, test-retest, and sensitivity to change analyses [62].

#### Freiburg questionnaire of physical activity (FQoPA)

For the assessment of health-related physical activity, exercise, and estimation of energy expenditure we will apply the FQoPA [63]. Participants will be asked to report the amount of time spent in different activities during the past 7 days (everyday activities) and past month (sport and recreational activities). Energy requirements (MET) for physical activities are provided with the FQaPA allowing calculation of total weekly energy expenditure (<15 MET*h/week = “not active enough”, 15–30 MET*h/week = “meets basic public health recommendations for physical activity”, >30 MET*h/week = “satisfactory active”) [63]. The FQoPA has shown high test-retest reliability after 14 days and 6 months [56]. Validity of the FQoPA has been shown by correlating physical activity data with maximum oxygen uptake (r = .422) [56].

### Tuning fork test

A graduated Rydel-Seiffer tuning fork (Martin, Tuttlingen, Germany) will be used for testing vibration intensity at the internal malleolus of the dominant leg. The participants will be instructed to lie at ease in a supine position in a quiet, comfortably warm room. The tuning fork will be applied as perpendicular as possible resting on its own weight with the arms of the fork swinging maximally. Once the two arms are swinging, the fork vibrates at 64 Hz. Triangles with an arbitrary scale on calibrated weights at the extremities of the arms allow assessment of vibration threshold. When the participant indicates that vibration is no longer perceived, the point of intersection on the arbitrary scale (0 minimum to 8 maximum) is read. The readings of three repeated tests will be averaged and considered the vibration threshold. Pestronk et al. [53] were able to show that the Rydel-Seiffer tuning fork has high inter- and intrarater reliability.

### Balance and strength / power assessment

The primary outcome measures will be balance and strength / power at baseline (pre-test), after the intervention (post-test) and 12 weeks after the intervention (follow-up). In general, balance assessment will be performed before strength / power assessment to reduce interfering effects of muscle fatigue [64].

### Balance assessment and gait analysis

Static steady-state balance will be assessed using the Romberg Test and Sharpened Romberg Test [65] while standing on a force platform (Leonardo 105 Mechanograph®, Novotec Medical GmbH, Pforzheim, Germany, measurement error: ≤0.2%). Participants will have to perform 4 tasks with increasing level of difficulty: (1) standing in an upright position with feet closed and eyes open for 10 s without swaying while holding both arms extended to the front with palms facing upwards; (2) ditto, but with eyes closed; (3) ditto, but eyes open and feet in tandem stand; (4) ditto, but eyes closed and feet in tandem stand. Centre of pressure (CoP) displacements in medio-lateral (CoP_ml_s_ in mm) and anterior-posterior (CoP_ap_s_ in mm) directions as well as standing time during the different test conditions will be assessed. Test termination criteria are displacing feet, lowering arms or opening eyes. Besides CoP displacements, stand time will be recorded using a stopwatch to nearest 0.01 s. Age-specific corresponding norm values are 14 s to 15 s (female) and 14.3 s to 17.5 s (male) [65]. For the Romberg Test (eyes open, ICC = .86 and eyes closed, ICC = .84) and Sharpened Romberg Test (eyes open, ICC = .70 and eyes closed, ICC = .91) high test-retest reliability has been shown [66].

Dynamic steady-state balance will be tested while walking on an instrumented 10-m walkway using a two-dimensional OptoGait^©^-System (Bolzano, Italy). Participants will walk with their own footwear at self-selected speeds, initiating and terminating each walk a minimum of 2 m before and after the 10-m walkway to allow sufficient distance to accelerate to and decelerate from a steady-state of ambulation across the walkway. The rectangular OptoGait^©^-System is an opto-electrical measurement system consisting of transmitting and receiving bars for obtaining a two-dimensional measurement area. Each bar is 1 m in length and contains 100 LEDs that transmit continuously to each other. With a continuous connection between the two bars, any break in the connection can be measured and timed. The walking pattern will be monitored at 1,000 Hz, enabling spatial and temporal gait data to be collected. The OptoGait^©^-System demonstrated high discriminant and concurrent validity with a validated electronic walkway (GAITRite^©^-System) for the assessment of spatio-temporal gait parameters in orthopedic patients and healthy controls [67]. Hausdorff et al. [68] reported that spatio-temporal parameters of gait are important mobility markers in community-dwelling older adults. Thus, means and standard deviations (SD) of stride time, stride length, stride velocity, and stride width will be computed. In addition, coefficients of variation (CV) for stride time, stride length, stride velocity, and stride width will be calculated according to the following formula: [CV = (SD / mean) × 100] [69]. Of note, the CV is a sensitive and clinically relevant marker for increased fall risk [70]. Further, it has been reported that gait velocity below 70 cm/s is associated with an increased risk of falling in old community-dwelling adults [71]. Thus, using a stopwatch to the nearest 0.01 s, gait velocity will be assessed as a marker of fall risk. Granacher et al. [72] recently reported that intraclass correlation coefficient (ICC) values for the above reported gait parameters were above 0.75. To resemble real life situations, static and dynamic steady-state balance will be tested under single (standing / walking) and dual task (standing / walking while counting backwards aloud) conditions. The cognitive interference task will comprise an arithmetic task, in which the participants recite out loud serial subtractions by three starting from a randomly selected number between 300 and 900 given by the experimenter [73].

Proactive balance will be assessed using the Functional Reach Test (FRT) [74] and the Timed up and Go Test (TUG) [75]. The FRT measures the maximal distance one can reach forward beyond arm’s length while maintaining a fixed base of support in the standing position. Maximal reach distance of the right and left arm will be recorded, whereas a distance between 15.4 cm to 25.4 cm indicates a moderate risk for falls [76]. The FRT will be measured while standing on a force platform (Leonardo 105 Mechanograph®) which additionally allows collection of CoP displacements. The FRT showed excellent test-retest reliability (ICC = 0.92) in older adults [74]. Validity of the FRT has been proved by Newton RA [77] when testing healthy community-dwelling older adults.

The TUG will be applied as described by Podsiadlo and Richardson [75]. Participants will be asked to perform the TUG at their self-selected habitual walking speed. One practice and one test trial will be performed. Time will be recorded with a stopwatch to the nearest 0.01 s. Before testing, a trained evaluator will provide standardized verbal instructions regarding the test procedures. Participants will be seated and instructed to walk 3 m, turn around, walk back to the chair and sit down. The stopwatch will be started on the command “ready-set-go” and stopped as the participant sits down. The TUG showed excellent test-retest reliability (ICC = 0.99) in older adults [75].

During the reactive balance test, participants will stand in bipedal step stance on a two-dimensional balance platform (Posturomed, Haider, Bioswing, Pullenreuth, Germany). The platform is mounted to four springs and is free to move in the transversal, ml, and ap directions. The maximal natural frequency of the Posturomed is below 3 Hz. The mechanical constraints and the reliability of the system were described earlier [78]. If the platform is in neutral position, the maximum range of motion in the ap and ml directions amounts to 70 mm, respectively. Medio-lateral perturbation impulses will be applied in order to investigate reactive postural control of the participants. Therefore, the platform will be moved 2.5 cm from the neutral position in the ml direction, where it will be magnetically fixed. For experimental testing, participants will be asked to stand (i.e., bipedal step stance) in erect position with hands placed on hips and gaze fixated on a cross on the nearby wall. Three to five trials help participants to get accustomed to the measuring device. After investigators visually control the position of the subjects, the ml perturbation impulse will unexpectedly be applied by detaching the magnet. The platform suddenly accelerates in the medial direction. The participants’ task is to damp the oscillating platform by balancing on the Posturomed. Summed oscillations of the platform in medio-lateral (SOml_r) and anterior-posterior (SOap_r) directions will be assessed by means of a joystick like 2D potentiometer (Megatron) which is connected to the platform. The potentiometer measures the position of the platform in degree [°]. The signal will be differentiated, rectified, and integrated over the 10 s test interval. Three trials will be performed. The best trial (least oscillations in ml direction) will be used for further analysis. Muehlbauer et al. [26] reported ICC values of 0.69 for SOml_r and 0.40 for SOap_r.

As a corresponding clinical test for reactive balance, the Push and Release Test (PRT) will be conducted. The PRT rates the postural response to a sudden release of a participant pressing backward on an examiner’s hands placed on a participant’s back [79]. The participant is instructed to stand in a comfortable stance with his or her eyes open and push backward against the palm of the examiners’ hands. After the examiner suddenly releases his or her hands, the participant is required to regain balance (backward stepping until a proper position is reached). During testing, the examiner will be responsible for safety of the participant. For rating purposes, the actual amount of steps to regain balance (not those to reorient the feet) will be measured (0 = 1 step, 1 = 2–3 small steps backwards with independent recovery, 2 = ≥4 steps with independent recovery, 3 = steps with assistance for recovery, 4 = fall or unable to stand without assistance). The PRT showed high test-retest reliability (ICC = 0.84) with a sensitivity of 89% and a specificity of 85%.

### Strength / power assessment

Handgrip strength will be measured to the nearest kilogram of each participant’s dominant hand using a Jamar hand dynamometer (Sammons Preston, Inc., Bolingbrook, IL) [80]. The dominant hand will be determined according to the lateral preference inventory [81]. The measurements will be performed with participants sitting in an upright position and with the arm of the measured hand unsupported and parallel to the body. The width of the dynamometer’s handle will be adjusted to each participant’s hand size so that the middle phalanges rested on the inner handle. We will instruct participants to exert maximal force. Starting with one submaximal trial to get accustomed to the testing procedure, participants will perform one maximal test trial. The intraclass correlation coefficient was calculated for handgrip strength (ICC = 0.99) [82]. Additionally, the Jamar hand dynamometer has been shown to have acceptable concurrent validity in young and adults [83].

Lower extremity strength / power will be assessed by the Chair Stand Test using a force platform (Leonardo 105 Mechanograph®) [84,85]. The Chair Stand Test will be performed as a clinical test, where participants will sit on a chair with their arms crossed on their chest, and stand up and sit down 5 times as quickly as they can. Time measured by a stop watch to the nearest 0.01 s indicates insufficient (≥16.7 s), sufficient (13.7 s to 16.6 s), good (11.2 s to 13.6 s), and very good strength performance (≤11.1 s) [85]. For the Chair Stand Test high test-retest reliability has been shown (ICC = .89) [86].

Participants will additionally perform maximal vertical countermovement jumps while standing on a force platform (Leonardo 105 Mechanograph®). The vertical ground reaction force will be sampled at 1,000 Hz. During the countermovement jumps, subjects stand in an upright position on the force plate and will be instructed to begin the jump with a downward movement, which will be immediately followed by a concentric upward movement, resulting in a maximal vertical jump. Subjects will perform three countermovement jumps with a resting period of 1 minute between jumps. For each of these trials, subjects will be asked to jump as high as possible. The best trial in terms of maximal jump height was taken for further data analysis. In a study by Granacher et al. [43] intraclass correlation coefficient was calculated for countermovement jumps power and amounted to ICC = 0.81.

The Stair Climb Power Test (SCP) will be used as a clinical equivalent for the countermovement jumps [87]. Participants will be instructed to safely ascend a 10-stair flight (each stair height 16.5 cm) as fast as possible. Use of the handrail will be allowed for safety reasons only. Timing begins after the countdown “ready-set-go” on the word “go” and stops when both of the participant’s feet reached the top step. Time will be measured by a stopwatch to the nearest 0.01 s and the average of 2 trials will be taken. SCP will be calculated by the formula: [power = force x velocity]. Test-retest reliability has been recorded and proved to be excellent (r = .99) [87].

### Assessment of body composition

A non-invasive bioelectrical impedance analysis (BIA) will be conducted before balance and strength / power assessments to minimize the effect of hydration status on measurements. Participants will be instructed to abstain from caffeine and alcohol 24 h, and exercise 12 h prior to testing according to published guidelines for BIA [88]. For BIA an octopolar tactile-electrode impedance meter (InBody 720, BioSpace, Seoul, Korea) will be used to estimate body composition according to the manufacturer’s guidelines. Multiple frequencies at 5, 50, 250 and 500 kHz will be used to measure intracellular and extracellular water separately. The participants will be measured under laboratory conditions standing barefoot on the device. With abducted arms 15° and legs 45° apart, they will hold a hand electrode with contact of all 10 fingers while the heels and forefeet will be placed appropriately on the foot electrode. Then an alternating current of 250 mA of intensity will be applied to measure impedance of arm, trunk and leg muscles. Whole-body resistance will be calculated as the sum of segmental resistance (right arm + left arm + trunk + right leg + left leg). The BIA with InBody 720 has been validated by dual-energy X-ray absorptiometry (R^2^ = 0.93) [89]. In normal and overweight adults multiple frequency BIA underestimated percentage of body fat within the precision of the BIA instrument (2%) [88,89].

### Design of exercise interventions

Study participants will be randomized (http://www.randomizer.org) with a gender ratio of 1:1 into 2 intervention groups (INT1 and INT2) and a control / waiting group (CON). The first intervention group (INT1) will conduct a 12-week exercise program according to the practice guide developed by the expert panel. The program consists of task-specific exercises for (1) static steady-state balance, (2) dynamic steady-state balance, (3) proactive balance, (4) reactive balance, and (5) strength as well as (6) power, especially for the lower extremities and the trunk muscles. Exercises will be performed 3 times per week on non-consecutive days, twice supervised for 45 min. (incl. 15 min. for warm-up and cool-down), and once at home for 30 min. individually. The second intervention group (INT2) follows the same exercise routine as the first intervention group (INT1), except that they perform a short version of the program called '3 times 3’. After a supervised introduction into the '3 times 3’ program, INT2 will individually train at home 3 times per week for 30 min. Each '3 times 3’ training session will consist of only one exercise within the 3 domains (static balance, dynamic balance, and strength). Quality and quantity of exercises will be controlled by weekly phone calls and a training log book. The control / waiting group will not participate in any form of training during the experimental period, but will receive the extensive supervised program after the experimental period. Pre and post assessment of all variables for all groups (INT1, INT2, CON) will be performed before and after the 12-week intervention period. Follow-up measurements 12 weeks after the intervention cessation will allow the assessment of detraining effects. Duration of a single assessment amounts to 90 min per participant.

### Intervention program

**Figure 2:**
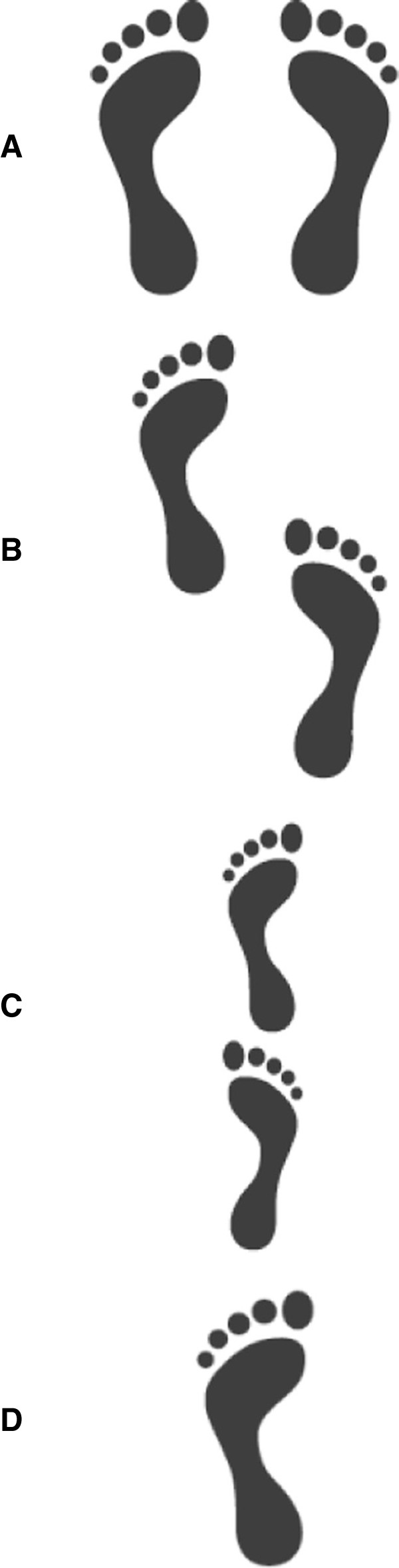
**Base of support during static steady-state balance. (A)** bipedal stance, **(B)** semi-tandem stance, **(C)** tandem stance, **(D)** monopedal stance.

**Figure 3:**
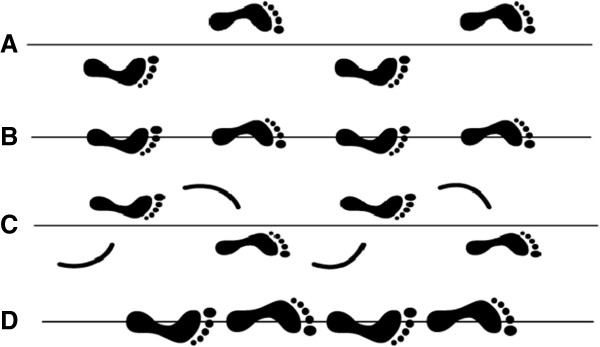
**Base of support during dynamic steady-state balance. (A)** normal gait, **(B)** narrow gait, **(C)** overlapping gait, **(D)** tandem gait.

**Figure 4:**
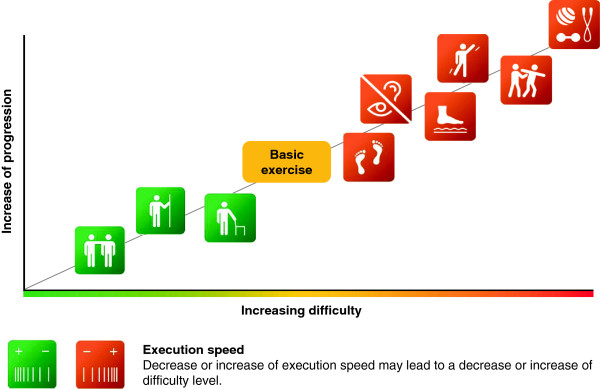
Exercise progression and variation during training.

The expert panel selected balance and strength / power exercises which can be performed with one’s own bodyweight or with the help of small, low-cost exercise equipment (i.e., small weights, resistance bands, unstable surfaces). However, intensity control for strength / power exercises performed with one’s own bodyweight is more complicated compared to when using strength training machines. In this study, intensity during training will be regulated using the Borg Rating of Perceived Exertion scale (i.e., 6–20 points, maximal exertion at 20 points) [90]. According to the individual fitness level, exercises should be performed with a perceived exertion between 10 and 16 points (light to hard) during balance and strength / power training. Exercise intensity will be progressed individually using the Borg Rating of Perceived Exertion scale and varying the balance and strength / power exercises in order to sufficiently stimulate the neuromuscular system [91]. Rate of perceived exertion will be adjusted every 2 weeks by the therapist (INT1) or via phone calls (INT2). Strength / power exercises will be progressed from single to multiple joint, isometric to dynamic muscle contraction, short to long lever arm and slow to fast exercises [92]. Further details regarding the contents of the intervention program are described in Tables 1 and 2 for strength / power training, and Tables 3 and 4 for static and dynamic steady-state, proactive, and reactive balance training (see also Figures 2, 3 and 4).

**Table 1:** Guidelines for heavy resistance strength training

**Exercise variables**	**Recommendations**
Intensity	Defined by level of difficulty, fatigue and number of repetitions
	Beginner: 12 – 13 RPE (somewhat hard)
	Advanced: 14 – 16 RPE (hard)
Quality	Technically correct movement
	Maximal range of motion
Speed of movement, contraction velocity	2 s concentric muscle contraction, 2 s eccentric muscle contraction (ratio 1:1)
Sets	2 – 3 (at home 3 sets)
Frequency	2 group sessions per week and 1 session alone at home (alternating strength / power and balance training)
Repetitions	Beginner: 10 – 15 (moderate resistance until muscle fatigue)
	Advanced: 8 – 12 (high resistance until muscle fatigue)
Rest	2 min. between sets

*RPE* rate of perceived exertion.

**Table 2:** Guidelines for muscle power training

**Exercise variables**	**Recommendations**
Intensity	Defined by level of difficulty, fatigue and number of repetitions
	10 – 13 RPE (light to somewhat hard)
Quality	Technically correct movement
	Maximal range of motion
Speed of movement, contraction velocity	Concentric contraction as fast as possible
	Approx. 1 s concentric muscle contraction, approx. 2 s eccentric muscle contraction (ratio 1:2)
Sets	2 – 3 (at home 3 sets)
Frequency	2 group sessions per week and 1 session alone at home (alternating strength / power and balance training)
Repetitions	8 – 10
Rest	2 min. between sets

*RPE* rate of perceived exertion.

**Table 3:** Guidelines for static steady-state, reactive, and proactive balance exercises

**Balance (static)**	**Exercise variables**	**Recommendations**
Steady-state	Base of support	Stable to instable: bipedal – semi-tandem – tandem – one leg stance (Figure 2)
	Position of feet	i.e., lateral or medial weight shift, on heels or toes, toe angle in or out
	Surface	i.e., from soft to hard (e.g., grass to concrete), from stable to instable (e.g., concrete to sand)
	Sensory input	Impede vision or hearing
	Dual-/Multi-tasking	Additional motor task – additional cognitive task – additional motor and cognitive tasks
	Speed of movement	Decrease or increase of execution speed (i.e., upper arm movements)
	Equipment	Use of i.e., free weights, elastic bands, balls
Reactive	Controlled perturbations applied by therapist	Reaction to external thread (push or pull) varying in speed, amplitude and direction on ankle, hip, trunk or shoulder level
Proactive	ADL	Combination of steady-state (static) balance tasks with mobility in daily life (e.g., standing up from a chair while reciting a poem and holding a cup of water)

*ADL* activities of daily living.

**Table 4:** Guidelines for dynamic steady-state, reactive, and proactive balance exercises

**Balance (dynamic)**	**Exercise variables**	**Recommendations**
Steady-state	Base of support	Stable to instable: normal gait – narrow gait – overlapping gait – tandem gait (Figure 3)
	Position of feet	i.e., lateral or medial weight shift, on heels or toes, toe angle in or out
	Surface	i.e., from soft to hard (e.g., grass to concrete), from stable to instable (e.g., concrete to sand)
	Sensory input	Impede vision or hearing
	Dual-/Multi-tasking	Additional motor task – additional cognitive task – additional motor and cognitive tasks
	Speed of movement	Decrease or increase of execution speed (i.e., walking speed)
	Equipment	Use of i.e., free weights, elastic bands, balls
	Direction	Forwards – backwards – to the left or right – diagonal
	Rhythm	Slow – fast – intermittent slow and fast
Reactive	Controlled perturbations applied by therapist	Reaction to external thread (push or pull) varying in speed, amplitude and direction on ankle, hip, trunk or shoulder level
Proactive	ADL	Combination of steady-state (dynamic) balance tasks with mobility in daily life (e.g., walking upstairs backwards while counting backwards aloud from 50 minus 2)

*ADL* activities of daily living.

### Statistics and sample size

An a priori power analysis was conducted to detect the sample size that is necessary to find statistically significant exercise effects [93] based on a study assessing the effects of balance training on postural control in older adults [94]. Considering a dropout rate of 10%, 18 participants per arm will be required to achieve 90% power (type II error of 0.10) with a type I error of 5%. Data will be analysed using a 2- and 3-way repeated measures analysis of variance (ANOVA) consisting of groups (INT1, INT2, CON) and time (pre-test, post-test, follow-up). Bonferroni post-hoc test will be used for statically significant (p < .05) group and time differences. Associations between clinical and biomechanical tests will be reported by their correlation coefficient (r value), level of significance (p value) and the amount of variance explained (r^2^ value). Values of r = 0.10 indicate a small, r = 0.30 a medium and r = 0.50 a large-size correlation [i.e., effect size] [95].

## Discussion

The nationwide implementation of effective fall prevention exercise programs in industrial countries is limited. The present trial applies and evaluates a public practice guide for balance and strength / power training that may provide a feasible, safe, and effective approach for fall prevention in older adults. In contrast to an epidemiological approach, in this trial, we will conduct an intervention based on three major intrinsic fall risk factors (balance impairments, gait instabilities, and muscle weakness). This will allow the use of several extensive clinical and biomechanical measurement tools for evaluation purposes. The proposed exercises require relatively low supervision and material costs, and offer practical information in terms of training volume, (i.e., type, frequency, duration) and intensity. A major advantage of this intervention compared to earlier fall prevention exercise programs is its broad and cost-free applicability and sustainability for German and French speaking older adults.

The expected effect of our fall prevention exercise program is based on a large recent meta-analysis by Gillespie et al. [12] who showed that multiple-component group exercise and home-based exercise reduce the rate of falls and fall risk (rate ratio 0.71, 95% confidence interval (CI) 0.63 to 0.82 and risk ratio 0.85, 95%CI 0.76 to 0.96 vs. rate ratio 0.68, 95%CI 0.58 to 0.80 and risk ratio 0.78, 95%CI 0.64 to 0.94). Previous studies showed that combined balance and resistance training may positively affect physical (i.e., balance and strength), mental (i.e., quality of life and fear of falling), and functional performance (i.e., ADL) [33,94,96-98]. Uncertainty remains if resistance training alone is sufficient to prevent falls in older adults [99]. Recent studies reported that especially muscle power exercises with lower loads and faster movement velocities improve ADL and therefore may be superior compared to traditional progressive resistance training [4,21,39,99-101]. In contrast, balance exercises are recommended for all older adults who had a fall [8], however, there is hardly any evidence about training load, volume, and frequency [21].

The current trial will add valuable information to the knowledge of dose–response-relations for exercise in older adults. Particularly the use of two different intervention arms (extensive supervised group exercise program vs. short home-based exercise program) will give some indication of the minimal amount of exercise needed to stimulate physical performance adaptations. If the short version of the program (3 times per week for 30 min.) will prove to be effective, this may lower the barrier for sedentary older adults to take up exercising. If intrinsic fall risk factors can be positively influenced by our proposed intervention regime, future trials will need to investigate any possible effect on fall rate in older adults. Additionally, in this trial, each clinical test will be compared to a gold-standard instrumented test. This cross-validation may facilitate the implementation of easy-to-administer balance and strength / power assessments into practice. Regular simple balance and strength / power assessments are important for training prescription and performance regarding exercise variation and progression. Furthermore, measuring gait velocity, grip strength and muscle mass will allow diagnosis of sarcopenia according to EWGSOP criteria, and may add knowledge to sensitivity and specificity of strength / power test to this important geriatric syndrome.

In summary, this trial will provide insight into the effect of fall prevention exercise applicable for a broad population and setting, both in community and sporting groups and at home. Practitioners, exercise therapists, and instructors will be provided with a feasible, validated exercise routine whose effect on intrinsic fall risk factors is scientifically evaluated. Furthermore, older adults who participate in the present program represent possible multipliers for a broader acceptance of important exercise and health-enhancing measures. Finally, the results of the current trial may help to further develop theories and models explaining balance and resistance training effects in general and particularly in older adults.

## Competing interests

The authors declare that they have no competing interests.

## Authors’ contributions

UG, YJG and BP were involved in the development of the fall prevention program. UG was responsible for the grant application for this trial. All authors contributed to the design of the study. UG and YJG wrote the paper, UG and BP will administer the fall prevention program. All authors read, critically revised, and approved the final version of the manuscript.

## Pre-publication history

The pre-publication history for this paper can be accessed here:

http://www.biomedcentral.com/1471-2318/13/105/prepub
